# LncRNA CYP4A22-AS1 promotes the progression of lung adenocarcinoma through the miR-205-5p/EREG and miR-34c-5p/BCL-2 axes

**DOI:** 10.1186/s12935-023-03036-z

**Published:** 2023-09-05

**Authors:** Liyao Dong, Lin Zhang, Xinyun Zhao, Hongling Zou, Sisi Lin, Xinping Zhu, Jili Cao, Chun Zhou, Zhihong Yu, Yongqiang Zhu, Kequn Chai, Mingqian Li, Qun Li

**Affiliations:** 1 College of Life Science, Sichuan Normal University, Chengdu, 610101 Sichuan China; 2 Zhejiang Provincial Key Laboratory of Cancer Prevention and Treatment Technology of Integrated Traditional Chinese and Western Medicine, Zhejiang Academy of Traditional Chinese Medicine, Tongde Hospital of Zhejiang Province, Hangzhou, 310012 Zhejiang China; 3 Hebei Province Key Laboratory of Research and Development for Chinese Medicine, Institute of Traditional Chinese Medicine, Chengde Medical College, Chengde, 067000 Hebei China; 4 People’s Liberation Army Joint Logistic Support Force 903th Hospital, Hangzhou, 330000 Zhejiang China

**Keywords:** CYP4A22-AS1, Lung adenocarcinoma, miR-205-5p, miR-34c-5p, EREG, BCL-2

## Abstract

**Objectives:**

Lung adenocarcinoma (LUAD) exhibits a higher fatality rate among all cancer types worldwide, yet the precise mechanisms underlying its initiation and progression remain unknown. Mounting evidence suggests that long non-coding RNAs (lncRNAs) exert significant regulatory roles in cancer development and progression. Nevertheless, the precise involvement of lncRNA CYP4A22-AS1 in LUAD remains incompletely comprehended.

**Methods:**

Bioinformatics analyses evaluated the expression level of CYP4A22-AS1 in lung adenocarcinoma and paracancer. The LUAD cell line with a high expression of CYP4A22-AS1 was constructed to evaluate the role of CYP4A22-AS1 in the proliferation and metastasis of LUAD by CCK8, scratch healing, transwell assays, and animal experiments. We applied transcriptome and microRNA sequencing to examine the mechanism of CYP4A22-AS1 enhancing the proliferation and metastasis of LUAD. Luciferase reporter gene analyses, west-blotting, and qRT-PCR were carried out to reveal the interaction between CYP4A22-AS1, miR-205-5p/EREG, and miR-34c-5p/BCL-2 axes.

**Results:**

CYP4A22-AS1 expression was significantly higher in LUAD tissues than in the adjacent tissues. Furthermore, we constructed a LUAD cell line with a high expression of CYP4A22-AS1 and noted that the high expression of CYP4A22-AS1 significantly enhanced the proliferation and metastasis of LUAD. We applied transcriptome and microRNA sequencing to examine the mechanism of CYP4A22-AS1 enhancing the proliferation and metastasis of LUAD. CYP4A22-AS1 increased the expression of EREG and BCL-2 by reducing the expression of miR-205-5p and miR-34-5p and activating the downstream signaling pathway of EGFR and the anti-apoptotic signaling pathway of BCL-2, thereby triggering the proliferation and metastasis of LUAD. The transfection of miR-205-5p and miR-34-5p mimics inhibited the role of CYP4A22-AS1 in enhancing tumor progression.

**Conclusion:**

This study elucidates the molecular mechanism whereby CYP4A22-AS1 overexpression promotes LUAD progression through the miR-205-5p/EREG and miR-34c-5p/BCL-2 axes.

**Supplementary Information:**

The online version contains supplementary material available at 10.1186/s12935-023-03036-z.

## Background

Lung cancer is a serious threat to human health and is the main cause of cancer-related deaths worldwide owing to its continued high incidence and mortality [1]. Since patients with lung cancer are asymptomatic in the early stages, they are usually diagnosed in the middle or late stages [2]. As cancer progresses, it metastasizes, and brain metastases are one of the primary causes of cancer-related deaths [3]. Even though past tremendous efforts targeting early diagnosis means and therapeutic methods have yielded remarkable progress, the current situation of lung cancer patients is not optimistic, with a five-year survival rate of only about 15% [4]. Based on its origin, lung cancer can be classified into small-cell lung cancer and non-small-cell lung cancer (NSCLC). Approximately 85% of lung cancer cases are NSCLC cases, and LUAD is the most common pathological type of NSCLC [5]. Accordingly, it is necessary to comprehensively explore the pathogenesis of LUAD to find sensitive biomarkers and clinical treatment strategies.

Cancer is usually attributed to an aberrant gene expression, essentially described as the aberrant expression of protein-coding and non-coding transcripts [6]. A study has reported that less than 2% of human genomes can encode proteins, and most of the remainder that are unable to encode proteins are identified as non-coding RNAs (ncRNAs) [7]. In the past, ncRNAs have been regarded as “useless” transcripts. However, several studies have revealed that ncRNAs control various physiological and pathological processes of various diseases, including cancers [8]. Long non-coding RNA (lncRNA) is a kind of ncRNA with a long nucleotide chain [9]. As a functional regulatory molecule, lncRNA has a specific and complex secondary space structure inside the molecule, which provides multiple sites for binding to proteins and interacts specifically with DNA as well as RNA through base complementary pairing [9]. LncRNAs are aberrantly expressed in a variety of cancers [10]. Compared with lncRNAs, microRNAs (miRNAs) have shorter nucleotide chains, which bind to the target messenger RNAs (mRNAs) to repress the translation or promote the degradation of target mRNAs [11–13]. Surprisingly, approximately 50% of miRNAs were located in the chromosomal regions associated with cancer [14]. MiRNAs, which are differentially expressed in normal and tumor tissues, can either induce or suppress the development of cancer [15, 16]. The interaction between lncRNAs and miRNAs has been found to influence tumor progression by regulating the proliferation, differentiation, and apoptosis of tumor cells [17]. On the one hand, lncRNAs serve as a competing endogenous RNA (ceRNA) to sponge miRNAs or compete with miRNAs for the 3 ‘UTR of the same mRNA, thereby inhibiting the regulation of mRNAs by miRNAs [18]. On the other hand, lncRNAs also act as miRNA precursors, which are cleaved by RNase III Drosha and Dicer to produce miRNAs, thus affecting mRNA expression [19]. In turn, miRNAs also target lncRNAs and play a negative regulatory role [20]. Therefore, the imbalance of lncRNA–miRNA interaction may lead to the occurrence and development of cancers, and more technical means are required to clarify how they interact with each other. Such advancements will help in clearly understanding the mechanism of cancer progression, ultimately providing new breakthroughs for cancer therapy.

LncRNA CYP4A22 antisense RNA 1 (CYP4A22-AS1), also known as ncRNA-a3, was identified as a conserved related lncRNA in the kidney and could stimulate TAL1 gene expression in MCF7 cells as an enhancer [21]. Increased CYP4A22-AS1 observed during extrathyroidal extension progression was associated with poorer disease-free survival in papillary thyroid carcinoma patients [22]. In addition, previous studies reported that CYP4A22-AS1 is involved in the prognosis of colorectal cancer and gastric carcinoma [23, 24]. Nonetheless, the mechanism of CYP4A22-AS1 in cancers is not well explicated.

In this study, we constructed a CYP4A22-AS1-overexpressed human LUAD cell line and found that the overexpression of CYP4A22-AS1 promoted cell proliferation and metastasis in LUAD. We further performed bioinformatics analysis and explored the molecular mechanism of its impact on LUAD progression. We found that a high expression of CYP4A22-AS1 could down-regulate miR-205-5p and miR-34c-5p to stimulate the increased expression of EREG and BCL-2 and activate the related signaling pathways, finally causing LUAD proliferation and metastasis. However, the transfection of miR-205-5p and miR-34c-5p mimics reversed the effect of CYP4A22-AS1 overexpression on LUAD. In brief, the findings of our study may provide new ideas and underlying therapeutic targets for the clinical treatment of lung cancer.

## Materials and methods

### Clinical samples message

The CYP4A22-AS1 expression of paired normal and tumor tissues in 48 LUAD patients was retrieved from the Cancer Genome Atlas (TCGA) data portal (https://portal.gdc.cancer.gov/repository).

### Cell culture

Human LUAD cell NCI-H1975 (cat. no. CL-0298) and human embryonic kidney cell HEK-293T (cat. no. CL-005) were purchased from Procell (Wuhan, China). The cell lines were cultured in the modified 1640 medium or Dulbecco minimal essential medium (DMEM, Biological Industries) with 10% fetal bovine serum (FBS, Biological Industries) under a humidified incubator containing 5% CO_2_ at 37℃. All cancer cells grew adherently. The cells in the logarithmic growth phase were selected for further experiments.

### Construction of the overexpression of CYP4A22-AS1 human LUAD cell

The CYP4A22-AS1 transcript (ENST00000444042.2) was cloned to PCDH-CMV-MCS-EF1-RFP-T2A-Puro vector. Recombinant lentiviruses were constructed using HEK-293T cells and packaging plasmids (PMD2.G and psPAX2). The recombinant lentivirus infected NCI-H1975 LUAD cells, and the cell monoclonal was screened with puromycin (10 µg/mL) to construct the CYP4A22-AS1 overexpressing cell line (H1975-CYP4A22-AS1, AS1 was used instead presented in the figures) and the empty vector cell line (H1975-PCDH, NC was used instead presented in the figures).

### RNA extraction and RT-qPCR

Total RNA was extracted using the Trizol method (Haoke, China), and cDNA was generated by a reverse transcription kit (Thermo, USA). The gene expression analysis was performed by reverse transcription-quantitative polymerase chain reaction (RT-qPCR) using an SYBR Premix Kit (Apibixo). Relative gene expression was quantified using the comparative threshold cycle (2^−ΔΔCt^) method. Sequences of the primers used in RT-qPCR (Qingke Biological Technology Services Co., Ltd, China) are shown in Supplementary Table 1.

### Cell counting kit (CCK-8) assay

The cells were digested with trypsin and cultured into a 96-well plate with 3000 cells/well for 30 wells in total and incubated for 0, 24, 48, 72, and 96 h. CCK-8 was added to each well with 10% final concentration at each point in time, and the OD values (450 nm) were measured after 2 h.

### Cell clone formation assay

After digestion with trypsin, the cells were plated in a six-well plate at a density of 300 cells/2 mL and then cultured in an incubator for 10 ~ 15 days. Next, methanol was used instead of the medium for 15 min, immediately washed off using phosphate-buffered saline (PBS), and the cells were stained with 0.1% crystal violet solution for 15 min. The crystal violet solution was washed off using PBS. The cells were observed, and images were collected under a microscope. Finally, the number of cells forming clones was determined.

### Transwell assay

We next used the transwell assay to assess cell migration and invasion. For the migration assay, 3.5 × 10^4^– 4 × 10^4^ cells were plated into the upper chamber with 200 µl serum-free 1640 medium, and 800 µl 1640 medium containing 10% FBS was added into the lower chamber. After 24 h of incubation, the chambers were removed and fixed with methanol for 15 min. After staining with 0.1% crystal violet (PBS) for 15 min, the cells in the chambers were wiped off with cotton swabs. The cells were observed, and images were collected under an inverted microscope. The number of cells passing through the chambers was determined. For the invasion assay, 30 µl 1:15 serum-free medium and Matrigel were added into the upper chamber and incubated for 2 h, and then the migration assay was repeated.

### Wound-healing assay

Cancer cells in the logarithmic growth phase were seeded into a six-well plate at a density of 4 × 10^5^ cells/well and covered with wound-healing experimental plug-ins. The plug-ins were placed in an incubator and cultured for 24 h. Plug-ins were removed, and the complete medium was replaced. Images were obtained at 0, 6, 12, and 24 h, and the area of the scratches was calculated using the Image J software. The wound-healing rate of each group relative to 0 h was compared. The wound-healing rate was calculated according to the following formula: healing rate = (initial trace area-final trace area)/initial trace area*100%.

### Western blot assay

The cells were seeded in a six-well plate at a density of 4 × 10^4^ cells/ well. Cell lysates were prepared using the RIPA lysis and centrifuged at 12,000 rpm for 15 min to separate the soluble components. Protein concentration was determined using a BCA protein detection kit in accordance with the manufacturer’s instructions with slight modifications. A solution of 10 µg/mL was prepared with the RIPA lysate, and the protein fragments were boiled in a metal sampler for 5 min. Samples containing 10–30 µg total protein were isolated on 4–12% SDS-PAGE gel. The protein was transferred from the gel to the nitrocellulose membrane (PALL) at a constant current of 300 mA for 90 min. The protein sample was sealed with 5% milk for 2 h at room temperature and incubated overnight with the corresponding primary antibody (diluted by 3% BSA) in an antibody incubator box. The protein sample was washed three times with TBST. Goat anti-rat and anti-rabbit IgG were used as secondary antibodies and incubated at room temperature for 2 h, then washed thrice with TBST. A chemiluminescence kit and chemiluminescence imager were used to detect the imprinting region. The ImageJ software was used to determine the area of the imprinting region. The antibodies are presented in Supplementary Table 2.

### RNA-seq and microRNA-seq analysis

The cells were seeded into a six-well plate at a density of 3 × 10^5^ cells/well and incubated for 12 h. Next, the cells were washed with PBS, and 800 µL TRIzol reagent was added to each well to dissolve. mRNA and miRNA library preparation was performed following the NEB common library construction method, and sequencing was performed at Novogene company laboratories. Differential genes were screened with DEseq2.

### Luciferase reporter assay

To find out the binding relationship between CYP4A22-AS1, EREG and miR-205-5p, the CYP4A22-AS1 (LUC-AS1-205: 5’-GGGGCCTGTTGGAGGGTGGGGGCTGGGAGGAG-3’) and EREG (LUC-EREG-205: 5’-TGGACAGTGCATCTATCTGGTGGACATGAGT-3’) sequenceswere respectively subcloned into pmirGLO vector (Qingke Biological Technology Services Co., Ltd, China). To find out the binding relationship between CYP4A22-AS1, BCL2 and miR-34c-5p, the CYP4A22-AS1 (LUC-AS1-34: 5’-TGCAGTGAGCCAAGATCGCGCCACTGCACTCCAGCCTG-3’) and BCL2 (LUC-BCL2-34: 5’-GAATCAGCTATTTACTGCCA-3’) were respectively subcloned into pmirGL vector. Next, according to the corresponding combination miR‑205-5p or miR‑34c-5p mimics was co‑transfected with CYP4A22-AS1 reporter vectors, EREG reporter vectors and BCL2 reporter vectors into 293 cells. The luciferase activity was investigated 24 h later by GloMax 20 (Promega Corporation).

### Transfection of miR-205-5p mimics or miR-34c-5p mimics with human LUAD cells

The cells were plated in a six-well plate at a density of 2.5 × 10^6^ cells/2 mL and incubated for 24 h. Next, miR-205-5p mimics (205 was used instead presented in the figures) and miR-34c-5p mimics (34c was used instead presented in the figures) were transferred into the H1975-PHCD and H1975-CYP4A22-AS1 cell lines at a concentration of 50nM, respectively, and cultured for another 24 h. Next, all cells were digested and further subjected to the CCK-8, clone formation, wound-healing, infiltration, and migration assays. For RT-qPCR and western blot analysis, transfection was performed similarly, and RNA or protein was extracted. H1975 cells were co-transfected with miR-205-5p/miR-34c-5p inhibitors at a concentration of 100nM using the same method. MiR-205-5P mimic/inhibitor (miR10000266-1-5/miR20000266-1-5) and miR-34c-5p mimic/inhibitor (miR10000686-1-5/miR20000686-1-5) were purchased from Ribobio Biotech Co., Ltd.

### Apoptosis of cancer cells

After digestion, the H1975 cells were seeded into a six-well plate as a system of 2 × 10^5^ cells/well. After incubation overnight, the medium was changed, and miR-205-5p and miR-34c-5p mimics were co-transfected into the H1975 cells and cultured for 24 h. Trypsin digestion was followed by centrifugation at 1,200 rpm for 5 min to collect dead and living cells. The cells were resuspended with 500 µL 1× AnnexinV Binding Buffer (prepared with deionized water) and transferred to flow tubes. The cells were stained with 5 µL PI and Annexin V-FITC for 15 min at room temperature in the dark. Flow cytometry was used to observe cell apoptosis. The FlowJO 7.6.1 software was used to analyze data and calculate the cell apoptosis rate.

### Xenograft mouse model

All animal studies were approved by the Animal Care Ethics Committee of the Zhejiang Academy of Traditional Chinese Medicine. Five male BALB/c nude mice weighing about 20 g were purchased from Zhejiang Academy of Medical Sciences. Each nude mice were subcutaneously injected with 5 × 107cells/100 µL H1975-PHCD (Left) and H1975-CYP4A22-AS1 (Right) cell suspension, respectively. After 45 days, the nude mice were sacrificed when the tumor size was obvious and the tumor tissues were collected for further analysis.

### Immunohistochemistry

For immunohistochemistry (IHC) analysis, deparaffinized graft tumor tissues were used. The sections were boiled in the sodium citrate buffer and incubated with primary antibodies p-AKT, p-ERK, and p-MEK at 4℃ overnight. Immunostaining was performed using 3,30-diaminobenzidine-tetrahydrochloride-dihydrate, and the samples were counterstained with hematoxylin. Negative controls were processed without the primary antibody. The nuclei were blue after hematoxylin staining, and the positive expression of DAB was brownish-yellow.

### Statistical analysis

For all in vivo and in vitro experiments, a two-tailed Student’s *t*-test was performed using GraphPad 8.0. The experimental data are reported herein as mean ± standard deviation (SD). Differences were considered statistically significant at p < 0.05, and the statistical significance was regarded as *p < 0.05 and **p < 0.01.

## Results

### High expression of CYP4A22-AS1 promotes the proliferation and metastasis of LUAD

**Fig. 1:**
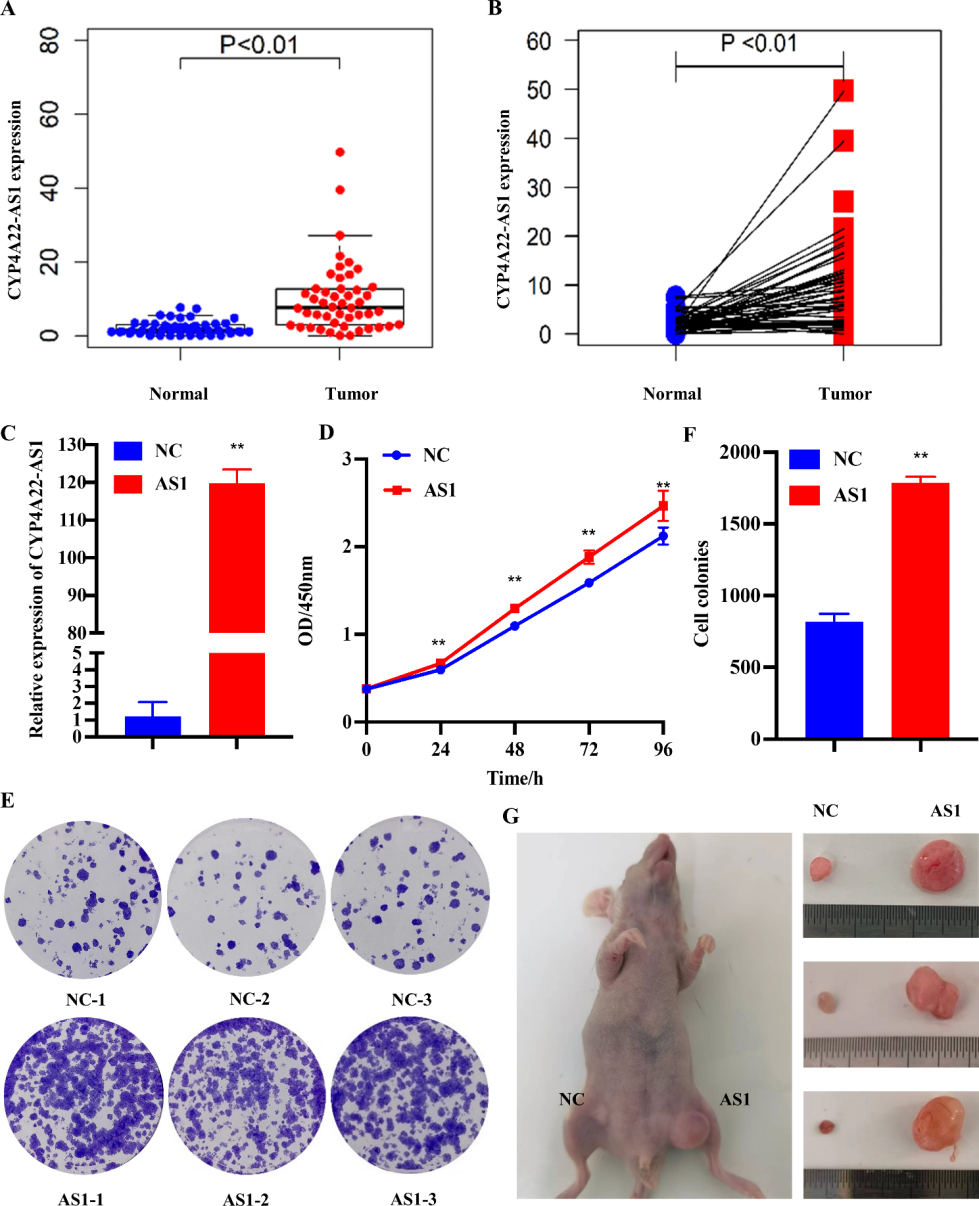
CYP4A22-AS1 is overexpressed in LUAD tissues and promotes LUAD cell proliferation in vivo and in vitro. **A** Online bioinformation analysis displayed that CYP4A22-AS1 was differentially expressed in LUAD tissues and normal tissues. **B** The expression of CYP4A22-AS1 in tumor tissues and adjacent tissues of LUAD patients was exhibited. **C** RT-qPCR was utilized for detecting CYP4A22-AS1 expression in the constructed LUAD cells. **D** Overexpression of CYP4A22-AS1 enhanced cell proliferation ability, as shown from CCK-8 assay. **E, F** The Cell clone formation assay further indicated that overexpressed CYP4A22-AS1 promoted cell proliferation. **G** The nude mice were injected with CYP4A22-AS1-overexpressed cells/control cells. Images of nude mice tumors revealed that overexpressed CYP4A22-AS1 promoted cell growth in vivo. GAPDH was used as the mRNA control. All of the experiments were performed thrice. **p < 0.01 demonstrated the statistical significance of the experimental data

**Fig. 2:**
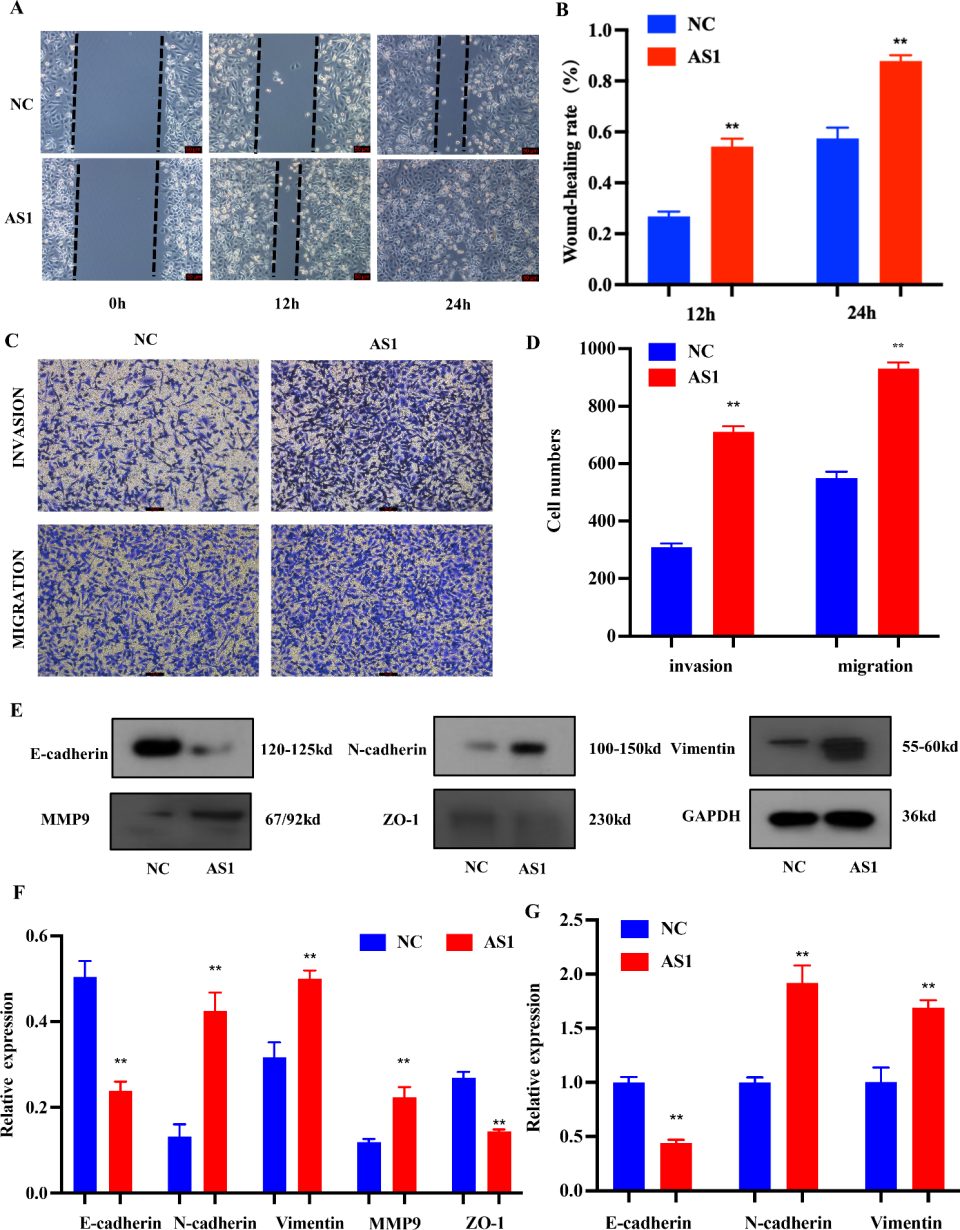
CYP4A22-AS1 activates the EMT pathway and promotes LUAD cell invasion and migration in vitro. **A, B** Cell migration capacity was tested with the wound-healing assay. Overexpressed CYP4A22-AS1 improved the cell migration capacity compared with that in the control group. **C, D** Transwell assays showed that cell invasion and migration abilities in high-expression CYP4A22-AS1 were increased, while those in the control group were decreased. **E, F** Western blot analysis of the expression level of related proteins of EMT in overexpressed CYP4A22-AS1 cells and in the control group. **G** RT-qPCR was adopted for measuring the mRNA levels of E-cadherin, N-cadherin, and Vimentin in LUAD cells. Compared with the low-expression group, the high expression CYP4A22-AS1 group up-regulated their expression levels. GAPDH was used as protein and mRNA control. All experiments were performed thrice. **p < 0.01 demonstrated the statistical significance of the experimental data

Through the analysis of the CYP4A22-AS1 expression in 48 LUAD patients from TCGA database, we found that the expression of CYP4A22-AS1 in tumor tissues was significantly higher than that in the adjacent tissues (p < 0.01; Fig. 1A, B). To investigate the role of CYP4A22-AS1 in LUAD, we constructed a CYP4A22-AS1 overexpressing cell line (H1975-CYP4A22-AS1) using a lentiviral expression system. The RT-qPCR results showed that the expression of CYP4A22-AS1 in H1975-CYP4A22-AS1 cells was significantly higher than that in H1975-PCDH cells (Fig. 1C). Furthermore, the CCK-8 cell proliferation assay revealed that a high expression of CYP4A22-AS1 could significantly improve the cell growth of H1975 cells (Fig. 1D). Consistently, clone formation experiments also demonstrated that CYP4A22-AS1 overexpression could accelerate cell growth in H1975 cells (Fig. 1E, F). To explore the effect of CYP4A22-AS1 in vivo, nude mice were injected subcutaneously with the H1975-CYP4A22-AS1 cells and corresponding H1975-PHCD cells. Mice were then randomly divided into three groups of control. After 45 days, mice were sacrificed, and tumors were removed for further analysis. As shown in Fig. 1G, the overexpressed CYP4A22-AS1 exhibited a stronger ability to promote cell growth compared with the control group. The wound-healing, infiltration, and migration assays further confirmed that a high expression of CYP4A22-AS1 could activate the EMT (epithelial-to-mesenchymal transition) signaling pathway. The results of the wound-healing assay showed that the overexpression of CYP4A22-AS1 discernibly enhanced cell motility in H1975 cells (Fig. 2A, B). As shown in Fig. 2C, D, a high expression of CYP4A22-AS1 allowed more cells to migrate and cross the chamber, showing stronger invasion and migration abilities. We conducted the western blot analysis of several vital proteins in the EMT pathway, among which epithelial cadherin E (E-cadherin) and zonula occludens-1 (ZO-1) decreased, while neural cadherin (N-cadherin), Vimentin, and matrix metalloproteinase (MMP9) were up-regulated in overexpressed CYP4A22-AS1 cells compared with the control (Fig. 2E, F). E-cadherin expression decreased while N-cadherin and Vimentin expression increased compared with the control group (Fig. 2G).

### CYP4A22-AS1 targets mir-205-5p and miR-34c-5p

**Fig. 3:**
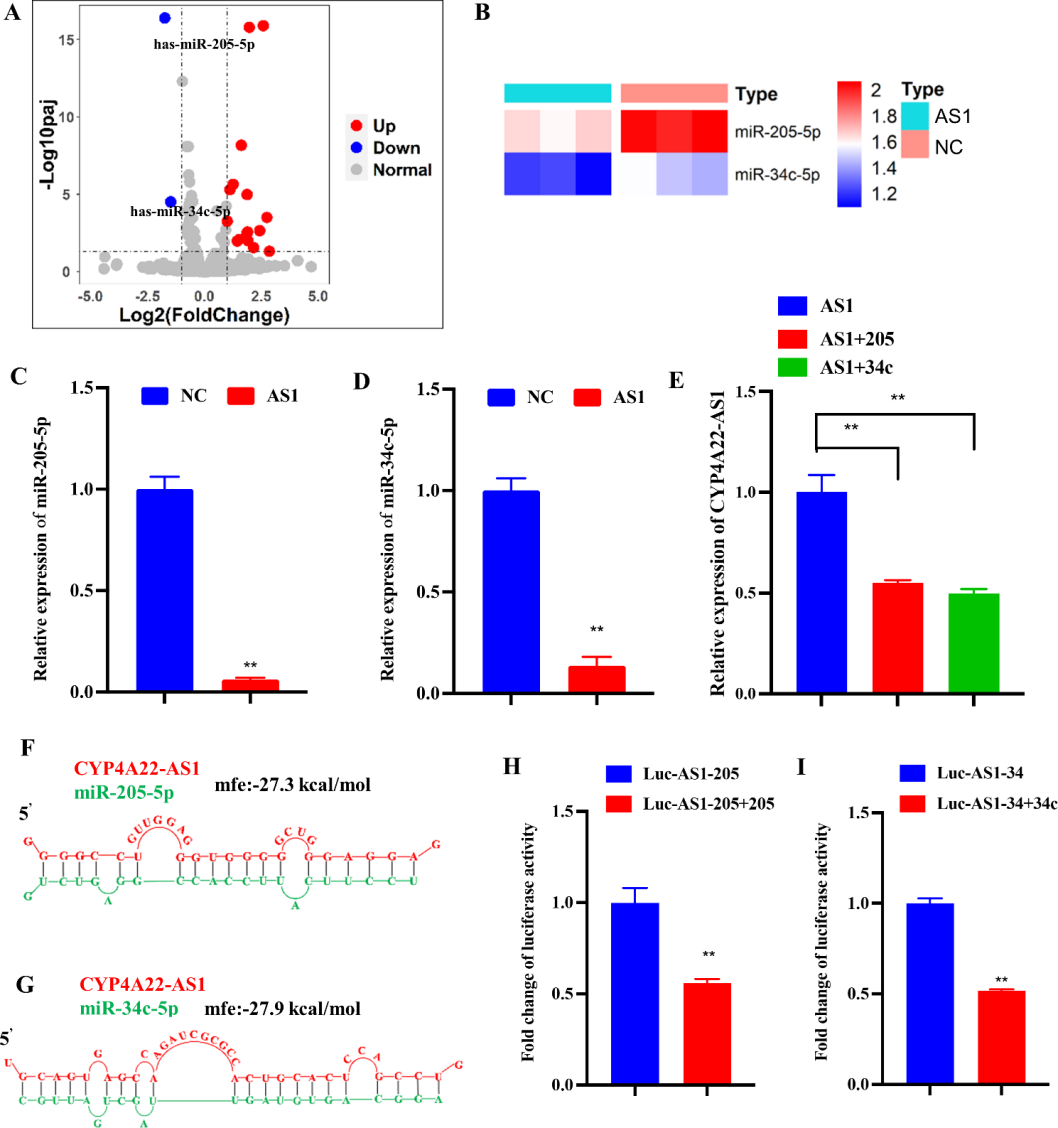
CYP4A22-AS1 directly targets miR-205-5p and miR-34c-5p. **A** Volcano plot presented the significantly down-regulated microRNAs in the high-expression CYP4A22-AS1 group compared with the control group (|Log2(fold-change)| > 1 and P.adj < 0.05). **B** The heat map exhibited the differential expressions of miR-205-5p and miR-34c-5p in overexpressed CYP4A22-AS1 cells and control.). **C, D** Differentially expressed microRNAs of CYP4A22-AS1-overexpressed cells were detected. CYP4A22-AS1-overexpressed cells discernibly reduced the expressions of miR-205-5p (C) and miR-34c-5p (D), while those in low-expression cells were elevated. **E** RT-qPCR was used to evaluate the expression of CYP4A22-AS1 in LUAD cells after co-transfection with miR-205-5p and miR-34c-5p mimics. After co-transfection, the expression of CYP4A22-AS1 was declined. **F, G** The diagram displays the miR-205-5p (F) and miR-34c-5p (G) binding sites with CYP4A22-AS1. **H, I** Luciferase reporter experiments were performed to determine the luciferase activity in 293 cells. GAPDH was used as the mRNA control. All experiments were performed thrice. **p < 0.01 demonstrated the statistical significance of the experimental data

To verify the spongeous role of CYP4A22-AS1 in regulating gene expression in LUAD progression, RNA-seq and microRNA-seq of H1975-PHCD and H1975-CYP4A22-AS1 cells were analyzed and displayed in the heat map and volcano map (Fig. 3A, B). MiR-205-5p and miR-34c-5p were noticeably down-regulated in H1975-CYP4A22-AS1 cells compared with H1975-PHCD cells. Consistent with that, the RT-qPCR results showed that the expression levels of miR-205-5p and miR-34c-5p were markedly decreased in H1975-CYP4A22-AS1 cells compared with those of H1975-PHCD cells (Fig. 3C, D). Next, H1975 cells were co-transfected with miR-205-5p and miR-34c-5p mimics, and the expression of CYP4A22-AS1 was investigated. The RT-qPCR results revealed that both miR-205-5p and miR-34c-5p mimics reduced the expression of CYP4A22-AS1 in the CYP4A22-AS1 overexpressing cells (Fig. 3E). We employed the BiBiServ tool and obtained the sequence information of CYP4A22-AS1 and miR-205-5p as well as miR-34c-5p binding (Fig. 3F, G). Meanwhile, to verify the binding correlation between CYP4A22-AS1 and miR-205-5p or miR-34c-5p, luciferase reporter vectors (Luc-AS1-205 and Luc-AS1-34) were co-transfected into 293 cells with miR-205-5p or miR-34c-5p mimics, and we found that miR-205-5p and miR-34c-5p could reduce the luciferase activities of Luc-AS1-205 and Luc-AS1-34, respectively (Fig. 3H, I). Therefore, these findings revealed that CYP4A22-AS1 targets miR-205-5p and miR-34c-5p in LUAD.

### MiR-205-5p and miR-34c-5p inhibited LUAD cell proliferation and metastasis by down-regulating CYP4A22-AS1

**Fig. 4:**
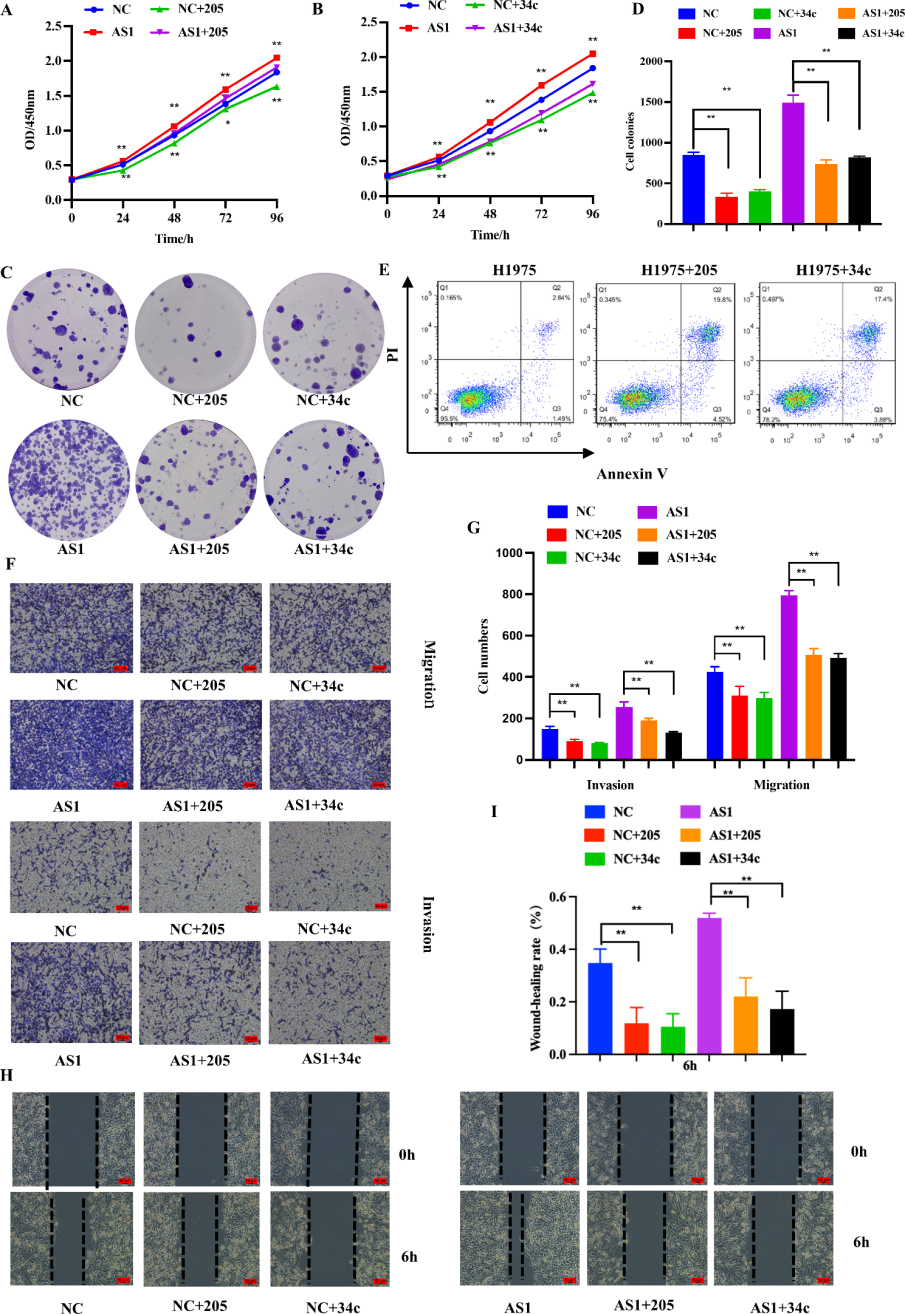
By down-regulating the expression of CYP4A22-AS1, miR-205-5p and miR-34c-5p inhibited LUAD cell proliferation, invasion, and migration in vitro. **A**, **B** Cell proliferation ability was examined using the CCK-8 assay after co-transfection with miR-205-5p and miR-34c-5p mimics. Both miR-205-5p and miR-34c-5p inhibited cell proliferation in overexpressed CYP4A22-AS1 cells. **C, D** The colony formation assay further demonstrated that transfection with miR-205-5p and miR-34c-5p mimics reduced the cell reproductive capacity in high-expression CYP4A22-AS1 cells. **E** Cell apoptosis rate was investigated after transfection with miR-205-5p and miR-34c-5p mimics in H1975 cells, as shown by the Annexin V assay. **F, G** Invasive and migratory abilities were detected by the transwell assay after co-transfection with miR-205-5p and miR-34c-5p mimics, respectively. **H** The wound-healing assay further showed that miR-205-5p and miR-34c-5p decreased the migration capability in overexpressed CYP4A22-AS1 cells. GAPDH was used as mRNA control. All experiments were performed thrice. *p < 0.05 and **p < 0.01 demonstrated the statistical significance of the experimental data

The H1975-CYP4A22-AS1 and H1975-PHCD cells were co-transfected with miR-205-5p and miR-34c-5p mimics, respectively, and the influence of miR-205-5p and miR-34c-5p on cell proliferation capacity was evaluated using CCK-8 and clone formation experiments. According to the results of the CCK-8 assay, both miR-205-5p and miR-34c-5p mimics reduced the proliferation ability of H1975 cells (Fig. 4A, B). The clone formation assay further indicated that miR-205-5p and miR-34c-5p mimics suppressed the growth of H1975 cells (Fig. 4C, D). In addition, H1975 cells were co-transfected with miR-205-5p and miR-34c-5p mimics, and cell apoptosis was detected. As shown in Fig. 4E, miR-205-5p and miR-34c-5p promoted the apoptosis of H1975 cells. Next, the infiltration and migration assays revealed that cell invasion and metastatic capacity were visibly decreased after transfection with miR-205-5p and miR-34c-5p mimics, respectively (Fig. 4F, G). Furthermore, the results of the wound-healing assay showed that both miR-205-5p and miR-34c-5p could inhibit the metastasis ability of H1975 cells (Fig. 4H, I).

**Fig. 5:**
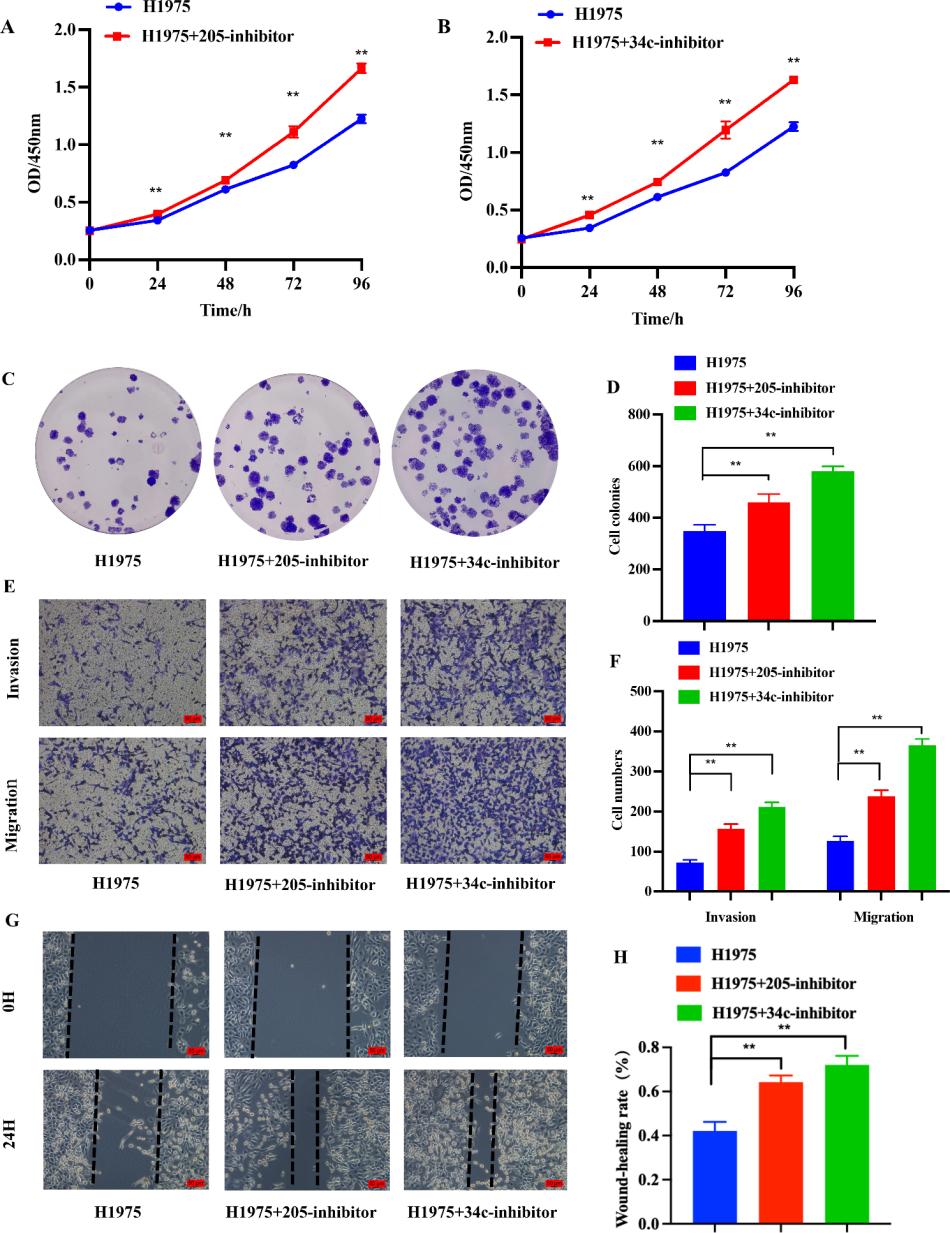
Inhibition of miR-205-5p and miR-34c-5p facilitates LUAD cell proliferation, invasion, and migration in vitro. **A, B** Enhanced H1975 cell proliferation capacity after co-transfection with miR-205-5p (A) and miR-34c-5p (B) inhibitors, as shown by the CCK-8 assay. **C, D** Clone formation assay showing that the proliferation ability was increased after co-transfection with miR-205-5p and miR-34c-5p inhibitors respectively in H1975 cells. **E, F** Transwell assays showing that invasion and migration abilities were improved in H1975 cells after co-transfection with miR-205-5p and miR-34c-5p inhibitors, respectively. **G, H** The wound-healing assay results of cell migration ability in H1975 cells after co-transfection with miR-205-5p and miR-34c-5p inhibitors. GAPDH was used as the mRNA control. All experiments were performed thrice. **p < 0.01 demonstrated the statistical significance of the experimental data

To verify the role of miR-205-5p and miR-34c-5p in H1975 cells, miR-205-5p and miR-34c-5p inhibitors were transfected into H1975 cells respectively. Subsequently, CCK-8 cell proliferation, clone formation, transwell, and wound-healing assays were applied to determine the ability of cell proliferation, invasion, and migration. Results from the CCK-8 assay, miR-205-5p and miR-34c-5p inhibitors distinctly promoted cell proliferation (Fig. 5A, B). Consistent with the results of the CCK-8 assay, the colony formation assay showed that cell growth was accelerated after transfection with miR-205-5p and miR-34c-5p inhibitors (Fig. 5C, D). Furthermore, the infiltration and migration assays revealed that miR-205-5p and miR-34c-5p inhibitors could improve cell invasion and migration abilities in H1975 cells (Fig. 5E, F). Also, the wound-healing assay showed that miR-205-5p and miR-34c-5p inhibitors enhanced cell motility capacity in H1975 cells (Fig. 5G, H). Altogether, both miR-205-5p and miR-34c-5p play important roles in inhibiting the progression of LUAD.

### MiR-205‐5p modulates the progression of LUAD by targeting EREG

**Fig. 6:**
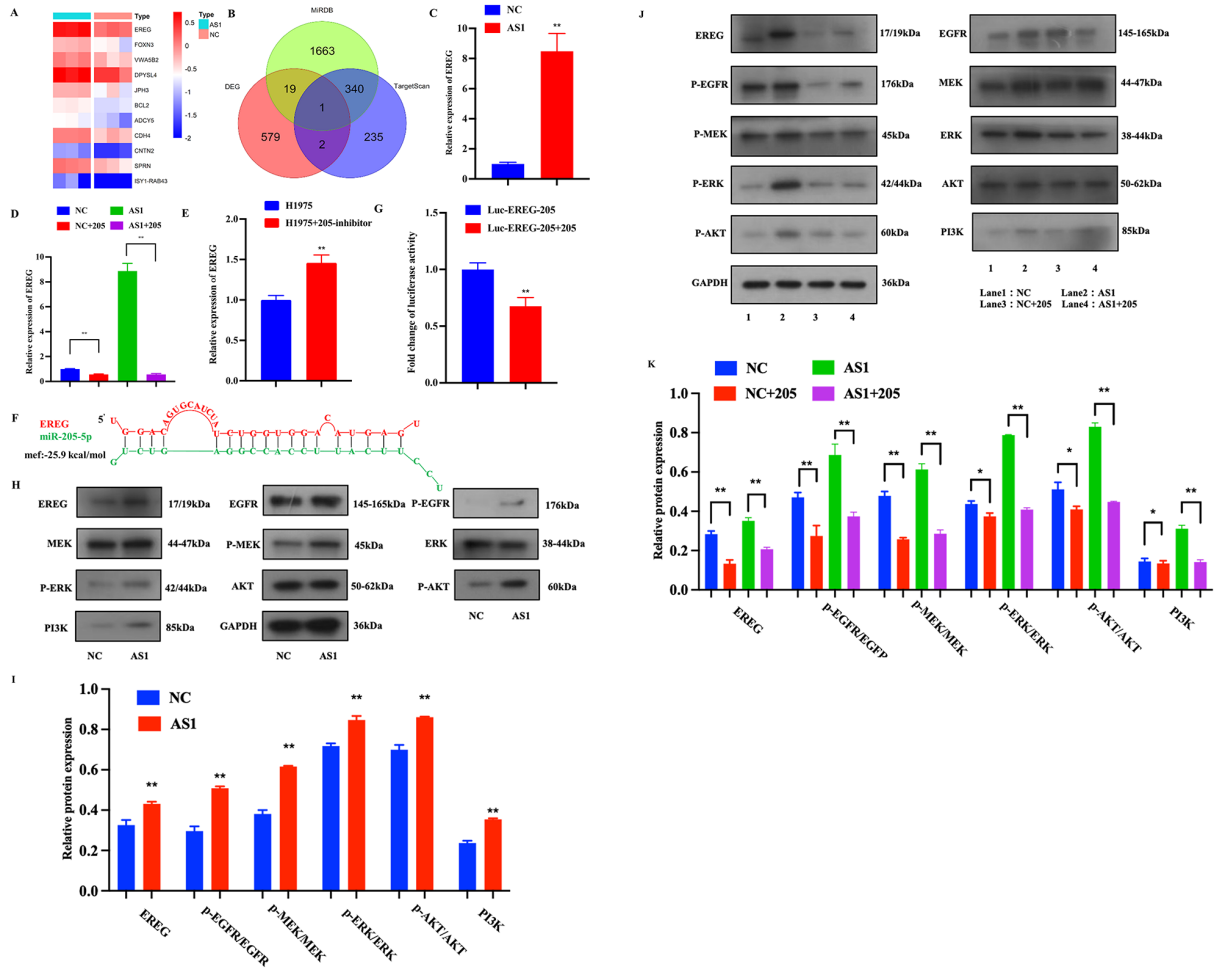
MiR-205-5p directly targets EREG in LUAD. **A** The heat map shows the differentially expressed genes between the overexpressed CYP4A22-AS1 group and control group. **B** Venn diagram shows a shared predicted target gene of miR-205-5p by three miRNA databases and overlapped with up-regulated ones induced by overexpressed CYP4A22-AS1. **C** RT-qPCR was employed to examine the expression of EREG. EREG was significantly up-regulated in the CYP4A22-AS1-overexpressed group compared with that of the control group. **D** After co-transfection with miR‑205-5p mimics, EREG was distinctly reduced in CYP4A22-AS1-overexpressed cells. **E** After co-transfection with miR‑205-5p inhibitors, EREG was up-regulated in H1975 cells. **F** The predicted binding sites of EREG with miR-205-5p. **G** The luciferase activity was remarkably reduced compared with that of the control group after transfection with miR-205-5p mimics in 293 cells, as shown by the luciferase reporter assay. **H, I** Western blot analysis of the expression of proteins involved in the EREG-activated EGFR downstream signaling pathway. In the CYP4A22-AS1-overexpressed group, EREG, P-EGFR, P-MEK, P-ERK, P-AKT, and PI3K were all increased. **J–K** After co-transfection with miR-205-5p mimics, EREG, P-EGFR, P-MEK, P-ERK, P-AKT, and PI3K were all significantly decreased. GAPDH was used as protein and mRNA control. All experiments were performed thrice. *p < 0.05 and **p < 0.01 demonstrated the statistical significance of the experimental data

MiRDB, TargetScan, and differential gene expression profiles were applied to find potential genes targeted by miR-205-5p. The result is presented in the heat map and the Venn diagram, showing that EREG was a potential target gene of miR-205-5p (Fig. 6A, B). Through the analysis of RT-qPCR, EREG was up-regulated in high-expression CYP4A22-AS1 cells (Fig. 6C). After the co-transfection with miR-205-5p mimics, the expression of EREG was investigated. As displayed in Fig. 6D, miR-205-5p mimics decreased the expression of EREG in over-expressed CYP4A22-AS1 cells. Moreover, miR-205-5p inhibitors were transfected into H1975 cells, and EREG was up-regulated, as per the results of the RT-qPCR analysis (Fig. 6E). The sequence information regarding miR-205-5p and EREG binding was obtained from the BiBiServ tool (Fig. 6F). Next, luciferase reporter vector (Luc-EREG-205) was constructed and co-transfected with miR-205-5p mimics into 293 cells, and the luciferase activity was measured after 24 h. The result showed that the relative luciferase activity was markedly reduced following co-transfection with miR-205-5p mimics (Fig. 6G). Furthermore, western blotting confirmed that the expression levels of EREG, P-EGFR, P-MEK, P-ERK, P-AKT, and PI3K, which are regarded as crucial proteins in the EGFR-related signaling pathways, were all increased in over-expressed CYP4A22-AS1 cells compared with the control group (Fig. 6H, I). In addition, we examined the expression levels of these proteins in H1975 cells after co-transfection with miR-205-5p mimics. The result, as expected, revealed a decrease in each protein’s level (Fig. 6J, K). Altogether, these findings, demonstrated that CYP4A22-AS1 could regulate EREG-stimulated EGFR-related signaling pathways by modulating miR-205-5p in LUAD.

### MiR-34c‐5p modulates the progression of LUAD by targeting BCL-2

**Fig. 7:**
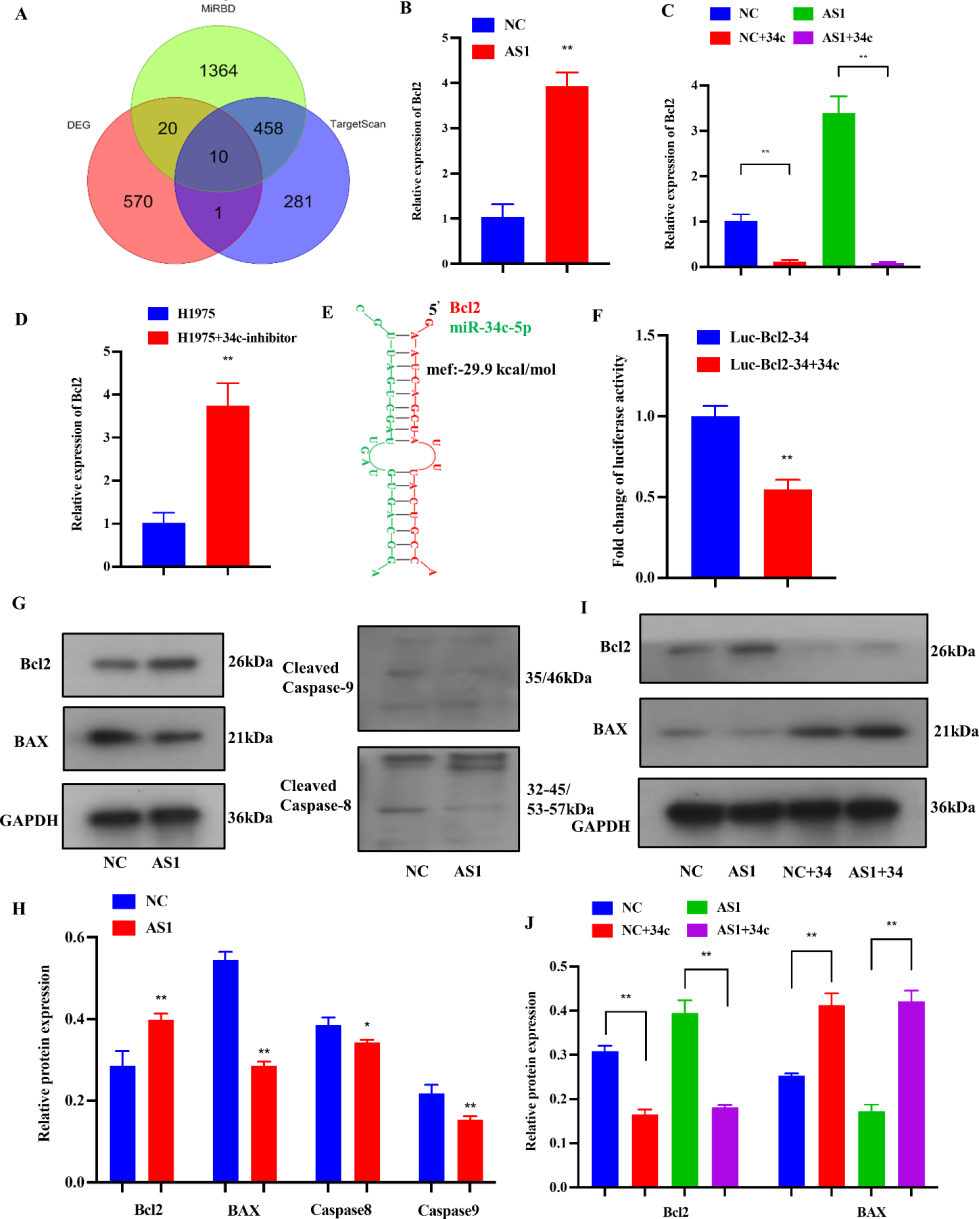
MiR-34c-5p directly targets BCL-2 in LUAD. **A** Venn diagram shows the shared predicted target genes of miR-34c-5p by three miRNA databases. **B** The expression of BCL-2 was measured with RT-qPCR. BCL-2 was up-regulated in the CYP4A22-AS1-overexpressed group compared with that in the control group. **C** After co-transfection with miR‑34c-5p mimics, BCL-2 was reduced in CYP4A22-AS1-overexpressed cells. **D** After co-transfection with miR‑34c-5p inhibitors, BCL-2 was increased in H1975 cells. **E** The predicted binding sites of BCL-2 with miR-34c-5p. **F** The luciferase activity markedly reduced compared with that of the control group after transfection with miR-205-5p mimics in 293 cells, as shown by the luciferase reporter assay. **G, H** Western blot analysis of the expression of proteins associated with apoptosis. Anti-apoptotic BCL-2 was increased, while pro-apoptotic BAX, caspase8, and caspase9 were decreased in the CYP4A22-AS1-overexpressed group compared with those in the control group. **I, J** After co-transfection with miR-34c-5p mimics, the expression of BCL-2 was decreased and BAX was increased, as shown by the western blot assay. GAPDH was used as protein and mRNA control. All experiments were performed thrice. *p < 0.05 and **p < 0.01 demonstrated the statistical significance of the experimental data

Likewise, we found 10 target genes of miR-34c-5p (Fig. 7A). Through the RT-qPCR analysis of these target genes, BCL-2 was found significantly up-regulated in high-expression CYP4A22-AS1 cells, consistent with transcriptome findings (Figs. S1 and 7B). Thus, BCL-2 was the prime study object of miR-34c-5p-targeted genes in this study, followed by miR-34c-5p mimics, which were co-transfected into H1975-CYP4A22-AS1 cells and H1975-PHCD cells. The RT-qPCR results showed that the expression of BCL-2 in CYP4A22-AS1 cells was decreased (Fig. 7C). Afterward, we co-transfected miR-34c-5p inhibitors into H1975 cells, and the expression of BCL-2 was increased, as demonstrated by the RT-qPCR analysis (Fig. 7D). To find out the binding site of BCL-2 to miR-34c-5p, the BiBiServ tool was used. Figure 7E presents the sequence binding information. After that, the luciferase reporter vector (Luc-Bcl2-34) was constructed and co-transfected with miR-34c-5p mimics into 293 cells, and as shown in Fig. 7F, the luciferase activity reduced in co-transfection with miR-34c-5p mimics group compared with the control group. Thus, to explore its molecular mechanism, we analyzed the expression of the BCL-2 signaling pathway-related crucial proteins by western blotting. The results revealed that CYP4A22-AS1 increased the expression levels of anti-apoptotic proteins, such as BCL-2, and decreased the expression levels of pro-apoptotic proteins, such as BAX, as well as two important pro-apoptotic execution proteases caspase8 and caspase9 (Fig. 7G, H). Simultaneously, we used western blotting to analyze the protein expressions of BCL-2 and BAX after being co-transfected with miR-34c-5p mimics, and the results showed that miR-34c-5p mimics exhibited exactly the opposite effect compared with before co-transfection in over-expressed CYP4A22-AS1 cells (Fig. 7I, J). Altogether, these results suggest that miR-34c‐5p affects the progression of LUAD by directly targeting BCL-2.

### CYP4A22-AS1 affects LUAD progression in vivo

**Fig. 8:**
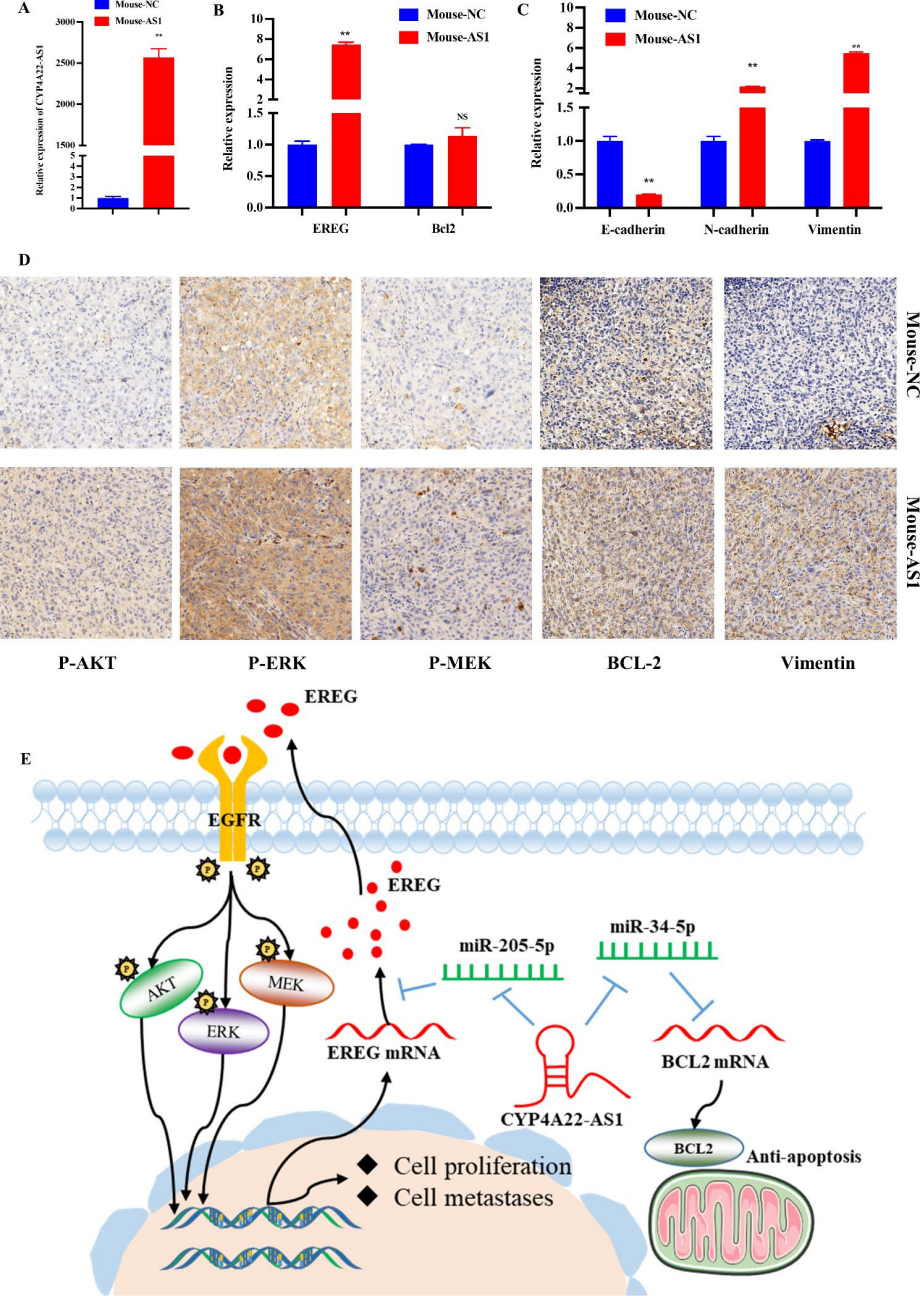
Effects of CYP4A22-AS1 on LUAD in vivo. **A** The nude mice tumors were removed for RT-qPCR. The expression of CYP4A22 was up-regulated in tumors of mice injected with CYP4A22-AS1-overexpressing cells. **B** As per the results of RT-qPCR, the expressions of EREG and BCL-2 were increased. **C** RT-qPCR was adopted to test the expression of markers of the EMT pathway in mice tumors. E-cadherin was decreased, while N-cadherin and Vimentin were increased in tumors of mice injected with CYP4A22-AS1-overexpressing cells. **D** IHC of p-AKT, p-ERK, p-MEK, BCL-2, and Vimentin in mice tumors, and their expression were all increased. **E** The underlying mechanism of CYP4A22-AS1 in LUAD. GAPDH was used as protein and mRNA control. All experiments were performed thrice. *p < 0.05 and **p < 0.01 demonstrated the statistical significance of the experimental data

The obtained mouse tumor cells were subjected to RT-qPCR to examine the expressions of CYP4A22-AS1, EREG, and BCL-2. Consistent with the results of in vitro experiments, these indicators were significantly differentially expressed in mouse tumor cells (Fig. 8A, B). Moreover, we also measured the expression levels of E-cadherin, N-cadherin and Vimentin in the EMT pathway, and the results were also consistent with our in vitro experiment results (Fig. 8C). The IHC assay demonstrated that the expression levels of p-AKT, p-ERK, p-MEK, BCL-2 and Vimentin were significantly increased in vivo after administration with H1975-CYP4A22-AS1 cells compared with H1975-PHCD cells (Fig. 8D). In summary, these findings suggest that CYP4A22-AS1 exhibit tumor-promoting effects through down-regulated miR-205-5p and miR-34c-5p targeting EREG and BCL-2 in vitro and in vivo (Fig. 8E).

## Discussion

Around 1.6 million people die from lung cancer every year, accounting for about 20% of cancer-related deaths, which are greater than breast, colon, and prostate cancers combined [25]. Lung cancer patients usually miss the optimal period of treatment owing to the lack of timely diagnosis, which leads to the deterioration of the disease [26]. LUAD is the most common type of lung cancer, accounting for about 40% of lung cancers, and often occurs in more advanced stages of the disease [27]. Hence, it is urgent to search for new biomarkers and therapeutic targets to improve the diagnosis and treatment of LUAD.

With the further research, ncRNAs, including lncRNAs and miRNAs, have been considered major players in cancer biology due to their involvemen in the development and progression of cancer [28, 29]. In this study, by establishing the lncRNA-miRNA-mRNA axis, we revealed that lncRNA CYP4A22-AS1 could as sponge to bind miR-205-5p and miR-34c-5p thereby regulating EREG and BCL-2 related signaling pathways and affecting the progression of LUAD. Although the relationship between CYP4A22-AS1 and LUAD has rarely been reported, we uncovered that CYP4A22-AS1 was significantly up-regulated in LUAD tissues through TCGA database and confirmed that CYP4A22-AS1 promoted the proliferation, metastasis and EMT of LUAD cells in vitro and in vivo. Notably, CYP4A22-AS1 expression was significantly up-regulated in mouse tumor cells compared to human LUAD cells, which warrants further exploration.

MiR-205-5p (formerly known as miR-205), a highly conserved and frequently silenced miRNA in cancer, has recently been implicated in the tumorigenesis and progression of numerous types of cancer. Many targets of miR-205-5p have been defined in cancer cells [30, 31]. Depending on the tumor and tissue type, miR-205-5p acts as either a tumor promoter or a tumor suppressor [32]. Notably, miR-205-5p was reported to be located in a lung cancer-associated genomic amplification region at 1q32.2 and participated in the occurrence and development of lung cancer, especially for NSCLC [33, 34]. Several previous studies have confirmed the cancer-promoting role of miR-205-5p in lung cancer, targeting genes such as PTEN, PHLPP2, and SMAD4 [35, 36]. However, our study revealed the anti-tumor effect of miR-205-5p in LUAD. Moreover, we demonstrated that miR-205-5p targets EREG to suppress LUAD cell proliferation and metastasis by blocking EREG-stimulated EGFR downstream signaling pathways. EREG, one of the ligands of EGFR, has a low expression in most normal tissues; however, in cancer, EREG up-regulates and activates EGFR [37]. Mutations and increased expression of EGFR are common in various cancers, among which lung cancer is the most common [38]. EGFR is at the front end of important signaling pathways in tumorigenesis, controlling multiple signaling pathways, such as PI3K/AKT and RAS/RAF/MEK/ERK, which drive cell proliferation and resist apoptosis [37]. Furthermore, activated EGFR regulates the PI3K/AKT and MEK/ERK signaling pathways to promote EMT, whereby epithelial cells are transformed into cells with a mesenchymal phenotype by a specific program, and it is closely related to the metastasis of tumor cells [39]. A single miRNA can indeed target multiple genes to affect their expression. Therefore, whether the miRNA promotes or inhibit cancer progression highly depends on the target genes [19]. This also explains why miR-205-5p can be both a “friend” and an “enemy” of cancers. We hope that our work will bring a new perspective to the study of the function of miR-205-5p in lung cancer.

MiR-34c-5p is a member of the miR-34 family, which can reduce cell proliferation and increase cell apoptosis [40]. Accumulating evidence indicates that miR-34c-5p is abnormally low expressed in common human cancers and is described a pivotal tumor suppressor in cancers [40]. Meanwhile, numerous target genes of miR-34c-5p have been identified in cancers, such as E2F3, BCL-2, and c-Met [41–43]. In our study, miR-34c-5p was shown to indeed regulate BCL-2 to play a tumor suppressor role in LUAD. The BCL-2 family of proteins, including apoptosis-promoting and apoptosis-inhibiting proteins, controls cell death primarily by regulating the direct binding of mitochondrial outer membrane permeability, leading to the irreversible release of intermembrane proteins, followed by caspase activation and apoptosis [44]. Indeed, differential gene expression analysis showed that BCL-2 expression was very low in LUAD cells, resulting in unstable expression, which may be why the difference in BCL-2 expression in mouse tumor cells was not significant.

However, we also found some miR-34c-5p target genes whose transcriptome was inconsistent with the RT-qPCR results, but the expression of some genes in high CYP4A22-AS1 high-expression cell lines was consistent with the regulated expression of miR-34c-5p inhibitors, such as JPH3, CDH4, DPYSL4, and ADCY5 (Figs. S1 and S2). Previously, JPH3 was identified as a novel methylated tumor-suppressor gene, which was shown to be a tumor suppressor gene in colorectal and gastric cancers [45]. CDH4 is responsible for encoding R-cadherin protein, which is pivotal in cell migration, adhesion, and tumorigenesis, and the expression of CDH4 in lung cancer tissues is significantly lower than that in adjacent tissues [46]. DPYSL4, a member of the collapse response regulatory protein family, is considered to be involved in tumor progression [47]. Mouse xenograft and lung metastasis models have shown that DPYSL4 expression inhibits tumor growth and metastasis in vivo [47]. ADCY5 (adenylate cyclase 5) is a member of the membrane-bound adenylate cyclase family, which converts adenosine triphosphate into the second messenger cyclic adenosine monophosphate and pyrophosphate and is regarded as a candidate diagnostic biomarker for colon cancer [48]. It follows that miR-34c-5p may target these genes to act on LUAD, and we conducted in-depth research on the role of these genes in LUAD under the regulation of miR-34c-5p and CYP4A22-AS1.

We illustrated that overexpressed CYP4A22-AS1 increased the expressions of EREG and BCL-2, which were regulated by miR-205-5p and miR-34c-5p, respectively, activated downstream signaling pathways, and eventually promoted the LUAD cell growth and metastasis. Moreover, cell biology and molecular biology experiment results revealed that the changes induced by CYP4A22-AS1 in LUAD cells could be reversed by miR-205-5p and miR-34c-5p through control of the targets genes and regulated the activation of downstream signaling pathways. Our study is anticipated to provide theoretical support for the development of new strategies for the treatment of LUAD.

### Electronic supplementary material

Below is the link to the electronic supplementary material.

Supplementary Material 1

## Data Availability

The datasets supporting the conclusions of this article are available from the authors upon reasonable request. If reasonable, the information about this study is available from corresponding author.

## Abbreviation

LncRNA: Long non-coding RNA
LUAD: Lung adenocarcinoma
miR-205-5p: MicroRNA-205-5p
miR-34c-5p: microRNA-34c-5p
EREG: Epiregulin
BCL-2: B-cell lymphoma-2
SCLC: Small cell lung cancer
NSCLC: Non-small cell lung cancer
3′UTRs: 3′-untranslated region
mRNAs: Messenger RNAs
ceRNA: Competing endogenous RNA
TCGA: The Cancer Genome Atlas
RT-qPCR: Reverse transcription quantitative polymerase chain reaction
CCK-8: Cell Counting Kit
ERK1/2: Extracellular response kinase ½
PI3K: Phosphoinositide 3-kinase
Akt: Protein kinase B
Caspase8: Cysteinyl aspartate specific proteinase8
Caspase9: Cysteinyl aspartate specific proteinase9
DMEM: Dulbecco Minimal Essential Medium
FBS: Fetal bovine serum
PBS: Phosphate buffer
IHC: Immunohistochemistry
GAPDH: Glyceraldehyde‑3‑phosphate dehydrogenase
CYP4A22-AS1: Cytochrome P450 4A22 antisense RNA 1
EMT: Epithelial-mesenchymal transition
EGFR: Epidermal growth factor receptor
MOMP: Mitochondrial outer membrane permeability
ETE: Extrathyroidal extension
PTC: Papillary thyroid carcinoma
MMP9: Matrix metalloproteinase 9
ZO-1: Zonula occludens 1
PHLPP2: PH domain and leucine rich repeat protein phosphatase 2
PTEN: Phosphatase and tensin homolog deleted on chromosome ten
RNaseIII: RibonucleaseIII
