# Supplementary material for: LncRNA CYP4A22-AS1 promotes the progression of lung adenocarcinoma through the miR-205-5p/EREG and miR-34c-5p/BCL-2 axes
Source: Cancer Cell Int. 2023 Sep 5;23:194. doi: 10.1186/s12935-023-03036-z (PMC10478502; doi:10.1186/s12935-023-03036-z)
Supplement: Supplementary file 1 — Supplementary Material 1 [file 12935_2023_3036_MOESM1_ESM.docx]

| **Supplementary Table 1. The primers of reverse transcription-quantitative PCR** | | |
| --- | --- | --- |
| Gene name Forward, 5'‑3' Reverse, 5'‑3' | | |
| CYP4A22-AS1 | GGGAGAGCATCAAGAATAGC | GTTCAAACGATTCTCCTGCC |
| N-caderhin | CATCATCATCCTGCTTATCCTGT | GCTCTTCTTCTCCTCCACCTTCTT |
| E-caderhin | GGGGTCTGTCATGGAAGGTGC | GTAAGCGATGGCGGCATTGTA |
| EREG | TGTGAAGTGGGTTATACTGG | TCGATTTCTGTACCATCTGC |
| Bcl2 | GTTCGGTGGGGTCATGTGTG | AGCTCCCACCAGGGCCAAAC |
| ADCY5 | AGATGAAGATCGGGCTCAAC | AGGAAGTAGGTCATCATCTC |
| JPH3 | AACTGGCGAGCCTGCGGCTG | TGAGCAAGATCACCATGACC |
| ISY1 | GCGAAGGAAGAAAATGGAAC | TGGTGATGTCGTAGGCAAGG |
| SPRN | CTCCTCCCTGCGCGTGGCTG | ACGCCCGGTAGCTGTAGATG |
| VWA5B2 | CCTTCGACGAGTGGGAACTG | CGTTCGCTGGGCTGTAGCAC |
| CDH4 | TGGTGCCGCACCCAGGCGAC | TCGTTGAGGTAATCGTAGTC |
| CNTN2 | CGGCTATAAGATGCTGTACC | AATGACGGTGCCAGGGTGTG |
| DPYSL4 | GGTGATGGTGCCTGCCAAGC | GGAGAGAGAGGTGATGTTGG |
| FOXN3F | ACGCATCCCAGCACAAGAAG | GGTGATGTTATTCAAACAGG |
| Vimentin | AATCCAAGTTTGCTGACCTCTCTGA | GACTGCACCTGTCTCCGGTACTC |
| GAPDH | CACCCACTCCTCCACCTTTG | CGTTCGCTGGGCTGTAGCAC |
| **Notes**: Primers of miR-205-5p(MQPS0000798-1-200), miR-34c-5p(MQPS0001100-1-200) and U6 (MQPS0000002-1-100) were synthesized by Ribobio Biotechnology Co., LTD. | | |

| **Supplementary Table 2. Antibodies were used for western blot and immunohistochemistry** | | | |
| --- | --- | --- | --- |
| **Name** | **Dilution ratio** | **Source** | **Cat** |
| E-caderhin | 1:10000 | Proteintech | 60335-1-lg |
| EREG | 1:1000 | ABclonal | A16372 |
| EGFR | 1:10000 | Proteintech | 66455-1-lg |
| P-EGFR | 1:1000 | Affinity | AF3044 |
| ERK1/2 | 1:2000 | Proteintech | 16443-1-AP |
| P-ERK1/2 | 1:1000 | Affinity | AF1015 |
| MEK1/2 | 1:1000 | Proteintech | 60267-2-lg |
| P-MEK1/2 | 1:2000 | Affinity | AF8035 |
| AKT | 1:5000 | Proteintech | 10176-2-AP |
| P-AKT1/2/3 | 1:5000 | Affinity | AF0016 |
| PI3K-P85 | 1:10000 | Proteintech | 60225-1-lg |
| Bcl2 | 1:1000 | Affinity | AF6139 |
| BAX | 1:10000 | Proteintech | 50599-2-lg |
| Caspase8/P43/P18 | 1:800 | Proteintech | 60335-1-lg |
| Caspase9/P35/P10 | 1:800 | Proteintech | 66169-1-lg |
| N-caderhin | 1:5000 | Proteintech | 22018-1-AP |
| Vimentin | 1:10000 | Proteintech | 60330-1-lg |
| ZO-1 | 1:10000 | Proteintech | 21773-1-AP |
| MMP9 | 1:800 | Proteintech | 10375-2-AP |
| GAPDH | 1:5000 | Proteintech | 60004-1-lg |


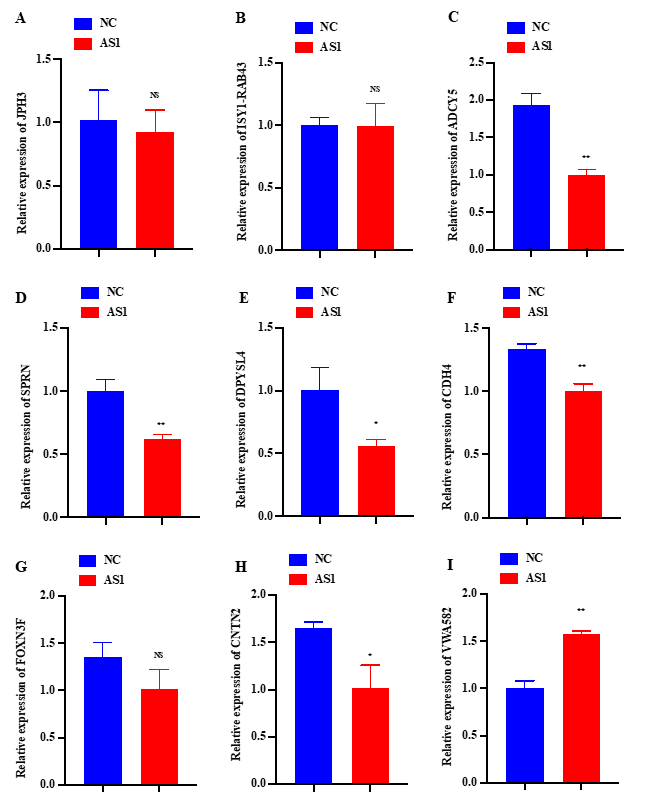


Figure S1. The expression of target genes of miR-34c-5p in LUAD cells.


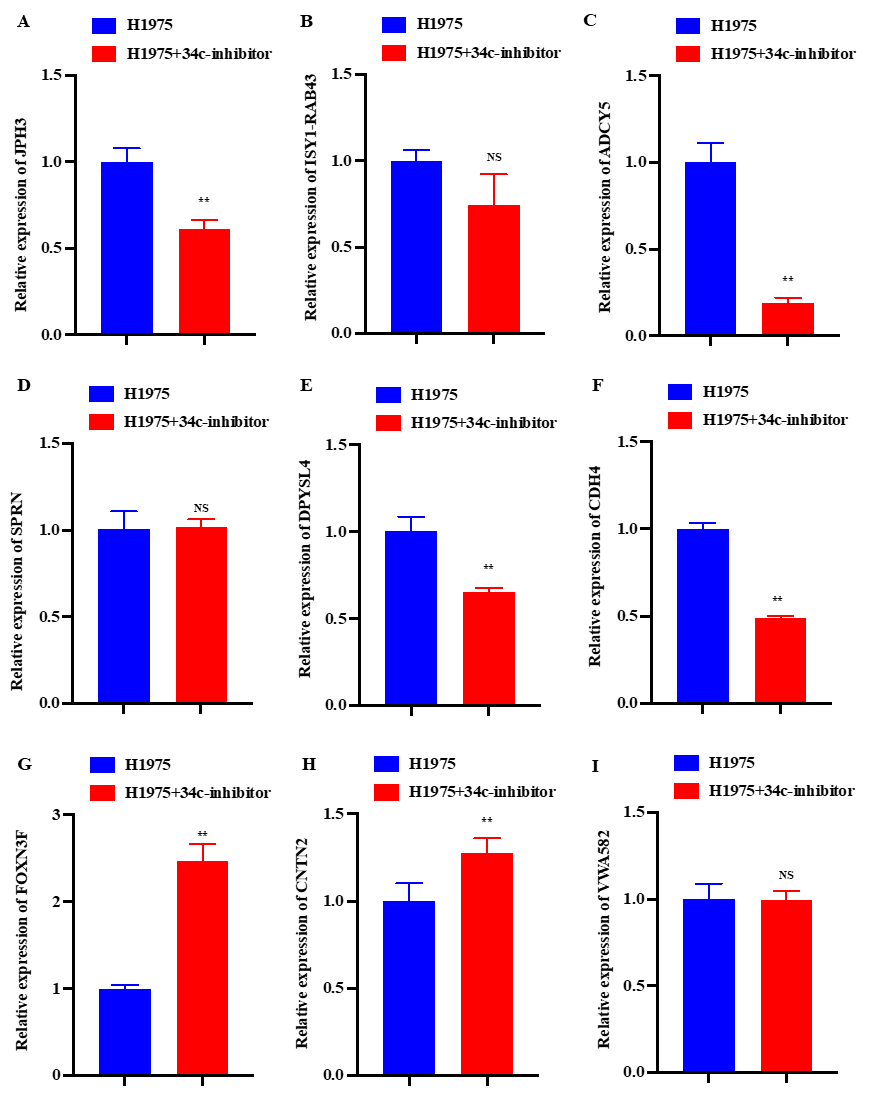


Figure S2. The expression of target genes of miR-34c-5p in LUAD cells after co-transfection with miR-34c-5p inhibitors.
